# Supporting Patients With Untreated Prostate Cancer on Active Surveillance: What Causes an Increase in Anxiety During the First 10 Months?

**DOI:** 10.3389/fpsyg.2020.576459

**Published:** 2020-11-19

**Authors:** Maria Francesca Alvisi, Paola Dordoni, Tiziana Rancati, Barbara Avuzzi, Nicola Nicolai, Fabio Badenchini, Letizia De Luca, Tiziana Magnani, Cristina Marenghi, Julia Menichetti, Villa Silvia, Zollo Fabiana, Salvioni Roberto, Valdagni Riccardo, Bellardita Lara, the Prostate Cancer Multidisciplinary Clinic Working Group

**Affiliations:** ^1^Prostate Cancer Program, Fondazione IRCCS Istituto Nazionale dei Tumori, Milan, Italy; ^2^Radiation Oncology 1, Fondazione IRCCS Istituto Nazionale dei Tumori, Milan, Italy; ^3^Department of Urology, Fondazione IRCCS Istituto Nazionale dei Tumori, Milan, Italy; ^4^Department of Oncology and Hemato-Oncology, University of Milan, Milan, Italy

**Keywords:** anxiety, active surveillance, prostate cancer, coping strategies, personality traits

## Abstract

**Background:**

The psychological burden possibly deriving from not immediately undergoing radical treatment for prostate cancer (PCa) could be a potential disadvantage of active surveillance (AS), especially in the eve of some relevant clinical exams [i.e., re-biopsy, prostate-specific antigen (PSA) test, and medical examination]. Even if it is known from the literature that the majority of PCa men in AS do not report heightened anxiety, there is a minority of patients who show clinically significant levels of anxiety after diagnosis. The present study aimed to investigate if demographic, clinical, and psychological variables at the entrance in AS (T0) were associated with the risk of developing clinically significant PCa-related anxiety 2 months before the first re-biopsy (T1) and to offer psychological support to improve quality of life (QoL).

**Materials and Methods:**

A total of 236 patients participated in the PCa Research International: AS (PRIAS) protocol and in PRIAS-QoL study. Demographic/clinical features, health-related QoL domains, coping with cancer, PCa-related anxiety [Memorial Anxiety Scale for PCa (MAX-PC)], personality traits, and decision-making-related factors were assessed at T0. MAX-PC was also administered at T1. PCa-related anxiety at T1 was considered to be of clinical significance if the MAX-PC score was ≥1.5. Multivariable logistic regression coupled to bootstrap was used to detect factors associated with high levels of anxiety.

**Results:**

The median age was 64.4 years. Fifty-six patients (24%) reported MAX-PC total score above the cutoff. Three factors were associated with a high level of PCa anxiety at T1: anxious preoccupation [odds ratio (OR) = 4.36], extraversion (OR = 1.9), and prostate-related symptoms (median OR = 0.46). Physical well-being was associated with a low PCa anxiety subscale (median OR = 0.15); neuroticism and functional well-being were associated with PSA anxiety (median OR = 7.05 and 0.73, respectively). Neuroticism and helplessness/hopelessness were associated with fear of progression (median OR = 18.1 and 5.8, respectively).

**Conclusion:**

Only a partial portion of the sample experienced significant levels of anxiety after 10 months. Psychological assessment should be routinely conducted to detect risk factors (i.e., anxious preoccupation, extraversion) for increased anxiety, offering tailored psychological interventions aimed at promoting interpersonal awareness and emotional well-being.

## Introduction

Active surveillance (AS) is increasingly considered a viable alternative to radical treatment (i.e., radical prostatectomy, external beam radiotherapy, or brachytherapy) for men with a diagnosis of very low/low-risk prostate cancer (PCa). Through systematic monitoring including repeated biopsies, digital rectal examination, repeated prostate-specific antigen (PSA) tests, AS offers the advantage to reduce overtreatment and safely delay or even avoid the risk of treatment-related side effects without losing the window of curability. From the patient’s perspective, it means to preserve one’s own health-related quality of life (HRQoL) at least as long as the monitoring does not show evidence that treatment is needed (Klotz et al., 2015; Bokhorst et al., 2016; Marenghi et al., 2017).

On the other side, a potential disadvantage of AS could be the psychological burden possibly deriving from not immediately undergoing radical treatment for PCa. Could living with “untreated” PCa cause anxiety in patients who chose AS? The available studies focusing on the assessment of HRQoL in AS patients showed that the majority of men did not report impairing anxiety. A small but even present distress may vary from the perceptions of health and overall psychological adjustment (Klotz et al., 2015). Nonetheless, there is a minority of patients who reported significant levels of anxiety after diagnosis, for example, in the run-up to clinical exams (i.e., re-biopsy, PSA test, and medical examination) (Anderson et al., 2014; Venderbos et al., 2015; Bokhorst et al., 2016; Parker et al., 2016). Those men showed a significant decrease of anxiety over time, which may suggest a relevant impact of coping strategies during the first period on AS (Marzouk et al., 2018; Dordoni et al., 2020). Anxiety and illness uncertainty were found to be predictors of HRQoL. Hence, interventions to reduce anxiety may enhance QoL for men with PCa on AS. Although anxiety is widely recognized as a central aspect that deserves attention in the AS population, little research has been conducted on anxiety-related factors (Bellardita et al., 2015). We found only two studies reporting associated factors of anxiety during AS. Neurotic personality and a higher level of PSA both assessed at the beginning of AS were shown to be associated with PCa-specific anxiety. In another study, intolerance of uncertainty was also suggested to promote anxiety on AS (Tan et al., 2016). Further studies are needed to understand the personal, clinical, and psychosocial features associated with anxiety during AS. Coping strategies are important factors during AS, since a good adjustment to cancer might be related to HRQoL and anxiety (Bellardita et al., 2015; Dordoni et al., 2020). Coping strategies are defined as constantly changing cognitive and behavioral efforts to manage specific external and/or internal demands (Folkman and Lazarus, 1984). Coping strategies are concerned with a person’s attempts to manage stressful circumstances, along with the ascribed meaning or interpretation given to such circumstances (Dordoni et al., 2020). Moreover, after diagnosis, men may enter a phase of “*decisional conflict*” (i.e., they feel uncertain about the course of action to be taken), which may increase their distress (Steginga et al., 2004; Bangma et al., 2013; Bellardita et al., 2018). This could guide the professionals managing patients on AS in developing effective interventions for the promotion of psychosocial well-being (Parker et al., 2016).

The aim of this study was to investigate which factors might predict a high level of PCa-related anxiety during the first 10 months on AS. Anxiety is supposed to be higher during the first 10 months of AS because the first re-biopsy (generally performed at 12 months after diagnosis) may disconfirm the observational option. Such follow-up could be seen as the first “turning point” and a critical moment for patients’ emotional well-being. The knowledge of variables affecting anxiety could be useful for both physicians (to offer focused psychological interventions at AS entrance) and to patients (to receive an even more “patient-centered care” aimed to prevent psychological burden during AS).

## Materials and Methods

### Procedures

This research is being conducted at the PCa Program of the National Cancer Institute in Milan since September 2007, with the aim of assessing the HRQoL over time and its associated factors for patients choosing AS. It was designed as single-center ancillary research to the multicenter prospective observational “PCa Research International: AS (PRIAS) study” (Bokhorst et al., 2016), in which selected men with low-risk PCa are managed based on a standardized protocol. Men were eligible for the PRIAS study if they had a diagnosis of PCa with a PSA <10.0 ng/ml; PSA density <0.2 ng/ml/cm^3^; clinical stage T1c or T2; ≤2 positive prostate needle-biopsy cores or <15% of positive cores in a saturation biopsy (≥20 total cores); Gleason Score of 3 + 3 = 6. To be considered a candidate for AS, a patient should be fit for radical treatment (i.e., radical prostatectomy, radiation therapy, or brachytherapy). The protocol involved PSA measurements every 3 months, physical examination every 6 months, and prostate biopsy at 1, 4, and 7 years after diagnosis and annually if the PSA doubling time was <10 years. Criteria for deferred active treatment are T stage >2, cancer in more than two cores at re-biopsies, or Gleason score >6. The scientific protocol and the related informed consent were approved by the local ethical committee.

### Sample

All patients with a PCa diagnosis included in the PRIAS protocol at our Institute were invited to participate in the ancillary QoL study (Ethical Commitee approved). Inclusion criteria for the study were (a) no evidence of mental disorders or cognitive impairments, (b) no evidence of physical conditions preventing the individual to read and fill in the questionnaire, (c) sufficient Italian language skill to understand the questionnaires, and (d) individual agreement with written informed consent.

Clinical/sociodemographic information was recorded at baseline. Self-report questionnaires evaluating HRQoL outcomes and other psychological variables were administered at different time points: at enrollment (T0) and 10 months after the diagnosis (i.e., about 2 months before the first re-biopsy) (T1). T0 administration was completed by patients when they signed the informed consent to enter PRIAS-QoL protocol. The follow-up questionnaires were sent by post or e-mail according to patients’ preferences. If the questionnaires were not returned within 1 month, patients received a reminder.

### Measures and Indicators

The anxiety specifically related to PCa was measured by the Memorial Anxiety Scale for PCa (MAX-PC) (Van Den Bergh et al., 2009) according to the Italian cultural adaptation for men in AS (Roth et al., 2003), which includes slightly modified subscales for PSA anxiety and fear of progression. Each of the 15 items is rated on a four-point Likert scale. Subscales and total score are calculated as mean values, thus ranging from 0 to 3, with 3 indicating maximum anxiety. It consists of 18 items divided into three subscales: (1) PCa anxiety, (2) PSA anxiety, and (3) fear of recurrence. The scale has been widely applied on samples of patients with PCa. Results showed that about 10% of patients report high levels of cancer-related anxiety (Roth et al., 2006).

Personality was assessed using the abbreviated form of the revised Eysenck Personality Questionnaire (EPQR-A), which consists of 24 items with two response options each (yes or no) (Alvisi et al., 2018). This tool provides three personality scales (Psychoticism, Extraversion, Neuroticism) and a control scale (Social Desirability).

HRQoL, i.e., the subjective perception about one’s own well-being and the extent to which it is affected by a medical condition, was assessed through the 36-Item Short Form Health Survey (SF-36) and the Functional Assessment of Cancer Therapy–Prostate version (FACT-P). The SF-36 (Francis et al., 1992) consists of 36 items that provide two summary scores: Physical Health (PH) and Mental Health (MH). Both total scores range from 0–100, with 100 indicating the best overall health. The FACT-P (Ware and Sherbourne, 1992) includes 39 items (four-point Likert scale) that assess different HRQoL dimensions: physical well-being, social well-being, emotional well-being, functional well-being, and PCa treatment-related symptoms. Scores were normalized based on the number of items included (score range: 0–4, with scores of 3–4 indicative of high well-being).

Adjustment to cancer, i.e., the coping style adopted to adjust to the cancer diagnosis, was evaluated through the Mini-Mental Adjustment to Cancer Scale (Mini-MAC). This scale (Esper et al., 1997) includes 29 items (score range: 1–4) measuring five different coping strategies, fighting spirit, helplessness/hopelessness, avoidance, fatalism, and anxious preoccupation, with higher scores indicating a greater presence of the specific coping style.

Decisional conflict, i.e., the patients’ perception of personal uncertainty about the choice of AS vs. the other feasible radical options was measured with the Decisional Conflict Scale (DCS) (Watson et al., 1994). This scale consists of 16 items with five response options (score range: 0–4): Informed, Values clarity, Support, Uncertainty, and Effective decision. Scale scores range from 0 (no decisional conflict) to 100 (extremely high decisional conflict).

Age, presence of a partner/spouse, education, and employment status were collected by an *ad hoc* survey at baseline.

Clinical data were collected from patients’ medical charts: the time between diagnosis and entrance in AS, the time gap between entrance in AS and T1, PSA at diagnosis, clinical stage, and positive/total cores at the diagnostic biopsy.

### Endpoints

The main endpoint of the study was PCa-related anxiety 2 months before re-biopsy at 1 year after diagnosis (T1). MAX-PC questionnaire was adopted to measure PCa-related anxiety. Clinically significance was defined by the following (Roth et al., 2003): scores ≥1.5 were considered as identifying high levels of anxiety. Total anxiety and the three specific subscales were considered as separated endpoints.

### Statistical Analyses

Associations between clinically significant anxiety and individual/clinical features were evaluated through Mann–Whitney test for each of the four endpoints. Multivariable logistic regression was performed to identify factors predicting high levels of anxiety. Variable selection was based on least absolute shrinkage and selection operator (LASSO) (O’Connor, 1995); missing values were imputed through flexible multiple imputations using bootstrapping (O’Connor, 1995) (completed data for Max-PC at T1 were available for 236 patients; regarding T0, some missing data for SF-36, Mini-MAC, EPQ-R, and FACT-P questionnaires were presented—24, 13, 68, and 18, respectively—and to solve this shortcoming, they were imputed). Bootstrap resampling (Tibshirani, 1996) (1,000 resamplings) was carried out for the evaluation of the odds ratios (ORs) of the selected variables to minimize the noise due to the particular dataset, thus trying to obtain an unbiased estimation of ORs. The performance of the resulting multivariable models was evaluated through calibration and Hosmer–Lemeshow test.

Statistical analyses were performed using MedCalc software version 12.1.4 (MedCalc Software, Mariakerke, Belgium), r-project,^1^ and KNIME software (KNIME GmbH, Germany).

## Results

### Patient Characteristics

Between September 2010 and May 2017, 449 patients were enrolled in PRIAS: 346/449 (77%) agreed to participate in the QoL study. Ninety-three (21%) refused, and 10 (2%) were excluded.

Complete data on MAX-PC at T0 and T1 were available for 236 patients. Table 1 shows the participants’ characteristics. Descriptive analyses for MAX-PC total score and subscales are reported in Table 2. Summary statistics for FACT-P, SF-36, EPQR-A, and Mini-MAC scores at T0 are available in the supplementary material.

**TABLE 1:** Patients’ characteristics.

	***N***	**%**
Total number of patients	236	
**Socio-demographic data**		
Age at diagnosis (years)	Median = 64.4	Range = 42–79
Higher education (High school)	155	66%
Employed	92	39%
Retired	138	58%
Missing	6	3%
Married or living with a partner	205	86.8%
Time between diagnosis and entrance in AS (months)	Median = 3.6	Range = 0–24.6
Time between entrance in AS and T1 (months)	Median = 6.4	Range = 0.5–9.9
PSA at diagnosis (ng/mL)	Median = 5.4	Range = 0.52–9.83
**Clinical stage**		
T1c	215	91%
T2a	21	9%
**Biopsy at diagnosis**		
1 Positive core	162	69%
2 Positive cores	74	31%

*AS, Active Surveillance, PSA, Prostate Specific Antigen.*

**TABLE 2:** Distribution of MAX-PC (Memorial Anxiety Scale for Prostate Cancer) total and subscale scores at T0 and T1.

***N* = 236**	**Mean**	**SD**	**Median**	**Observed score range**	***N* (%) of patients with clinically significant anxiety**
**T0 (*N* = 213)**					
MAX-PC total score	1.2	0.4	1.1	0.4–2.5	49 (23%)
PCa anxiety	0.8	0.6	0.7	0–2.6	34 (16%)
PSA anxiety	0.9	0.8	0.7	0–3	43 (20.2%)
Fear of progression	0.8	0.5	0.8	0–2.7	28 (13.1%)
**T1 (*N* = 236)**					
MAX-PC total score	1.2	0.4	1.1	0.5–2.2	56 (24%)
PCa anxiety	0.8	0.6	0.7	0–2.6	35 (15%)
PSA anxiety	1.1	0.8	1	0–3	72 (31%)
Fear of progression	0.9	0.6	0.8	0–2.7	38 (16%)

*SD, Standard Deviation; MAX-PC, Memorial Anxiety Scale for Prostate Cancer; PSA, Prostate Specific Antigen; N, number of patients; clinically significant anxiety: MAX-PC ≥ 1.5.*

### Factors Predicting the Risk of Higher Anxiety

At T1, 56/236 patients (24%) reported clinically significant anxiety as measured by total MAX-PC, 35 (15%) for PCa anxiety, 72 (31%) for PSA anxiety, and 38 (16%) for fear of progression. Considering 213 patients who completed both T0 and T1 assessments, significant changes between low and high levels of score (under and above clinical threshold, respectively) between T0 and T1 were observed: 9.4% for total MAX-PC, 4.7% for PCa anxiety, 16% for PSA anxiety, and 8% for fear of recurrence (*p*-values < 0.001, chi-squared).

Univariate associations between MAX-PC at T1 and FACT-P, SF-36, EPQR-A, Mini-MAC, and SCD are reported in Table 3.

**TABLE 3:** Associations between FACT-P (Functional Assessment of Cancer Therapy–Prostate), SF-36 (Short Form Health Survey-36 items), EPQR-A (Eysenck Personality Questionnaire), Mini-MAC (Mini-Mental Adjustment to Cancer Scale) and DCS (Decisional Conflict Scale) total and subscale scores at T0 and MAX-PC (Memorial Anxiety Scale for Prostate Cancer) total and subscale scores at T1 (Median scores and Mann-Whitney *p*-value).

	**MAX-PC total median**			**PCa anxiety median**			**PSA anxiety median**			**Fear of progression median**		
	**Low**	**High**	***p***	**Low**	**High**	***p***	**Low**	**High**	***p***	**Low**	**High**	***p***
**MINI-MAC**												
Fighting spirit	2.8	2.8	0.807	2.8	2.8	0.129	2.8	2.8	0.245	2.8	2.8	0.859
Helplessness/hopelessness	1.1	1.3	0.007	1.1	1.7	0.0001	1.1	1.3	0.015	1.1	1.7	<0.001
Avoidance	2.3	2.8	0.0005	2.3	3.0	0.0001	2.3	2.5	0.036	2.3	2.8	0.024
Fatalism	2.3	2.3	0.602	2.3	2.3	0.569	2.3	2.4	0.428	2.3	2.5	0.706
Anxious preoccupation	1.9	2.3	<0.0001	1.9	2.6	<0.0001	1.9	2.1	<0.0001	1.9	2.6	<0.001
**FACT-P**												
Physical well-being	4.0	4.0	0.11	4.0	3.9	0.007	4.0	4.0	0.497	4.0	3.9	0.005
Social well-being	2.9	2.6	0.023	2.9	2.5	0.007	2.9	2.7	0.222	2.9	2.5	0.003
Emotional well-being	3.3	3.0	<0.0001	3.3	2.8	<0.0001	3.3	3.0	<0.0001	3.3	2.8	<0.001
Functional well-being	2.7	2.4	0.0058	2.7	2.3	0.0001	2.7	2.4	0.035	2.7	2.3	0.001
Prostate symptoms	3.3	3.2	0.037	3.3	3.0	0.003	3.3	3.3	0.299	3.3	3.2	0.014
**SF-36**												
Mental health	52.4	48.5	0.012	52.6	45.3	0.0001	53.1	48.7	0.009	53.2	43.9	<0.001
Physical health	54.0	54.5	0.594	54.4	52.8	0.07	54.0	54.4	0.698	54.1	53.7	0.729
EPQ												
Psychoticism	0.2	0.2	0.31	0.2	0.2	0.91	0.2	0.2	0.568	0.2	0.2	0.877
Extraversion	0.7	0.5	0.931	0.7	0.5	0.841	0.7	0.5	0.366	0.7	0.5	0.306
Neuroticism	0.2	0.3	0.016	0.2	0.3	0.022	0.2	0.3	0.0007	0.2	0.5	<0.001
**DCS**												
Informed	25.0	25.0	0.223	25.0	25.0	0.31	25.0	25.0	0.726	25.0	25.0	0.323
Values clarity	25.0	25.0	0.877	25.0	25.0	0.729	25.0	25.0	0.774	25.0	25.0	0.719
Support	25.0	25.0	0.434	25.0	25.0	0.318	25.0	20.8	0.876	16.7	25.0	0.121
Uncertainty	25.0	25.0	0.252	25.0	37.5	0.039	25.0	25.0	0.106	25.0	37.5	0.006
Effective decision	25.0	25.0	0.196	25.0	25.0	0.167	25.0	25.0	0.54	25.0	28.1	0.062
Total decisional conflict	25.0	25.0	0.249	25.0	28.9	0.101	25.0	24.2	0.761	25.0	28.1	0.05

*FACT-P, Functional Assessment of Cancer Therapy-Prostate; SF-36, Short Form Health Survey; EPQ, Eysenck Personality Questionnaire; Mini-MAC, Mini-Mental Adjustment to Cancer; DCS, Decisional Conflict Scale; MAX-PC, Memorial Anxiety Scale for Prostate Cancer. Low, patients with MAX-PC values below cut-off for clinical significance (1.5), High, patients with MAX-PC values above cut-off for clinical significance.*

Presence of anxiety was positively associated with helplessness/hopelessness, avoidance, anxious preoccupation, FACT-P, MH, and neuroticism. Results for the four multivariable models are reported in Table 4.

**TABLE 4:** Logistic regression models for MAX-PC (Memorial Anxiety Scale for Prostate Cancer) total score, PCa anxiety subscale, PSA anxiety subscale and fear of progression subscale.

	**Median coeff**	**% Significant coeff**	**Median odds ratio**	**10° – 90° Percentile for ORs**
Endpoint: MAX-PC total ≥1.5				
Criterion: probability >0.20				
Sensitivity = 0.81, specificity = 0.66				
Hosmer-Lemeshow test, *p* = 0.95				
Calibration slope = 0.87, *R*^2^ = 0.96				
Extraversion	0.64	81.6	1.9	0.74 – 4.3
Anxious preoccupation	1.47	100.0	4.4	3.0 – 6.5
Prostate symptoms	−0.77	96.4	0.46	0.25 – 0.79
Constant	−2.12			
Endpoint: PCa anxiety ≥1.5				
Criterion: probability >0.14				
Sensitivity = 0.38, specificity = 0.85				
Hosmer-Lemeshow test, *p* = 0.90				
Calibration slope = 1.04, *R*^2^ = 0.89				
Physical well-being	−1.87	99.4	0.15	0.04 – 0.39
Constant	5.45			
Endpoint: PSA anxiety ≥1.5				
Criterion: probability >0.26				
Sensitivity = 0.76, specificity = 0.57				
Hosmer-Lemeshow test, *p* = 0.91				
Calibration slope = 0.90, *R*^2^ = 0.85				
Neuroticism	1.95	99.8	7.05	2.9 – 16.0
Functional well-being	−0.32	89.5	0.73	0.51 – 1
Constant	−0.58			
Endpoint: Fear of progression ≥1.5				
Criterion: probability >0.08				
Sensitivity = 0.93, specificity = 0.52				
Hosmer-Lameshows test, *p* = 0.94				
Calibration slope = 1.06, *R*^2^ = 0.97				
Neuroticism	2.89	100	18.1	6.4 – 57.1
Helplessness/Hopelessness	1.76	100	5.8	3.1 – 11.7
Constant	−5.17			

*Coeff, coefficient; % significant coeff, percentage of coefficients >0 if mean coefficient is >0; percentage of coefficient <0 if mean coefficient <0; ORs, Odds Ratios; MAX-PC, Memorial Anxiety Scale for PCa, PCa, Prostate Cancer, PSA, Prostate Specific Antigen.*

Three factors resulted as predictors of high MAX-PC total score at T1: the EPQR-A extraversion (median OR = 1.9) and Mini-MAC anxious preoccupation (median OR = 4.36) were associated with an increased risk of anxiety, while FACT-P subscale PCa symptoms had a protective effect (median OR = 0.46).

Physical well-being at the entrance in AS was associated with PCa-related anxiety at T1 as a protective factor (median OR = 0.15). High PSA-related anxiety scores were associated with EPQR-A neuroticism subscale and to FACT-P functional well-being (median OR = 7.05 and 0.73, respectively).

Anxiety for fear of progression resulted in a two-variable model: neuroticism and helplessness/hopelessness from Mini-MAC (median OR = 0.18 and 5.83, respectively).

**FIGURE 1:**
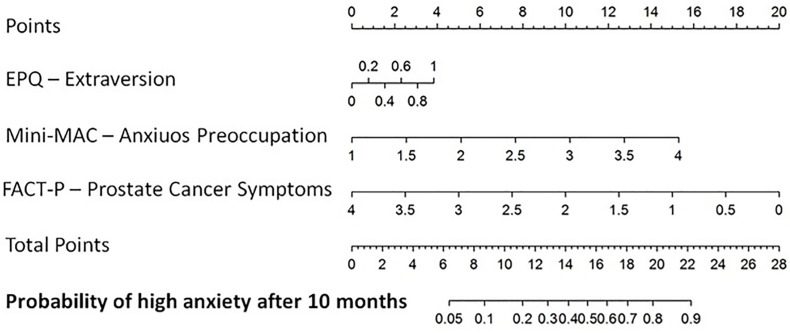
Nomogram for the probability of having Memorial Anxiety Scale for Prostate Cancer total score >1.5 after 10 months from the diagnostic biopsy. Extraversion (continuous variable) of EPQR-A, Anxious preoccupation (continuous variable) of Mini-MAC, Prostate cancer-related symptoms.

Calibration plots for all the models are presented in the supplementary material. Figure 1 reports the nomogram derived from the total MAX-PC logistic model presented in Table 4.

## Discussion

Even though clinical exams (i.e., re-biopsy, PSA test, and medical examination) may influence patients’ anxiety and psychological well-being, scarce studies have investigated patients on AS’ HRQoL in the run-up to clinical exams. This study offered information on anxiety levels among PCa patients in AS in proximity of the first clinical examination and on factors predicting anxiety. Clinicians should be aware of how patients on AS live critical moments of AS protocol (i.e., clinical exams) and of what impacts on this, so to support men preventing psychological burden from the very beginning of entrance in AS.

Our investigation confirms that only a partial portion of PCa patients on AS experience troubling levels of anxiety (24% of patients reported the MAX-PC total score above the threshold). These results are consistent with previous studies focusing on PCa-related anxiety (Roth et al., 2003; Anderson et al., 2014; Venderbos et al., 2015; Bokhorst et al., 2016). Specifically, we here identified PSA-related anxiety as particularly relevant with up to 31% patients reporting distress when dealing with PSA testing. Anxiety indeed is a key component of such burden (Van Buuren, 2012). It is well known that PSA may be considered a “red flag” for PCa patients (Palorini et al., 2016).

Our findings also suggested that personality traits, coping strategies, and perceptions of functional well-being were associated with anxiety.

Within personality traits, the extraversion trait, i.e., indicating a talkative and lively person, who likes/enjoys meeting new people (Alvisi et al., 2018), was found to be a risk factor in predicting anxiety (Villa et al., 2017). This may seem counterintuitive. Yet we can argue that extravert men may more easily show anxiety compared to introvert men, since they are more confident in communicating their feelings (Villa et al., 2017). Such sharing could turn out to be useful, as communicating their anxiety may help them ask for emotional support.

Neuroticism (i.e., characterizing an irritable person, who is often troubled about feelings of guilt and whose mood often goes up and down) increases PCa-related anxiety (Klotz, 1997; Villa et al., 2017). Men who scored high on neuroticism were usually preoccupied about the potential progression of the disease, reacting emotionally to monitoring events (Jylhä and Isometsä, 2006). Particularly, our results revealed that neuroticism traits were associated with PSA-related anxiety and fear of progression. Men with more neurotic personalities were also previously found to have a higher chance of anxiety (Jylhä and Isometsä, 2006; Riggio and Riggio, 2002).

Helplessness/hopelessness coping strategies emerged as risk factors for anxiety (Bellardita et al., 2013; Villa et al., 2017). Such strategies refer to the perception that an individual has not enough resources and support to cope with stressful events. As a consequence, men may experience a lack of control resulting in both anxiety and little likelihood of engaging in a healthy lifestyle (Dordoni et al., 2020).

Additionally, our results showed that men who were coping with high anxious preoccupation at AS enrollment were more likely to experience overall cancer anxiety after 10 months. Coping strategies (measured through the Mini-MAC questionnaire) have shown a relevant impact on cancer patients’ emotional well-being and on men on AS’ quality of life (Dordoni et al., 2020). Psychological interventions directed on problem-focused coping (i.e., strategies used in situations valuated as controllable) and emotion-focused coping (i.e., strategies used in situations in which nothing can be done) may help patients in engaging in healthier behaviors and reduce cancer-related anxiety (Bellardita et al., 2013; Dordoni et al., 2020).

PCa symptoms, physical well-being, and functional well-being had a protective effect on anxiety in our sample (see mental and physical health on FACT-P results). Men on AS usually do not report specific PCa-related symptoms; however, it is likely that those who enter AS perceiving few cancer-related symptoms take their minds off cancer, resulting in positive emotional well-being. Note that the FACT-P PCa symptoms scale in the case of men in AS is indeed measuring prostate-related symptoms mainly due to benign prostatic hyperplasia.

Finally, in the present study, no sociodemographic nor patient-related data were significantly associated with higher anxiety levels. Anderson et al. (2014) reported a limited role for sociodemographic data in explaining patients’ anxiety during AS. Only a few variables such as being divorced and age have been found to impact on patients’ well-being.

To monitor patients’ anxiety level and prevent a potential psychological burden, a specific tool supported by clinical data was needed. For this purpose, in the present study, logistic regression model was translated into a specific prognostic nomogram, which could sustain the entire care process supporting clinicians in foreseeing patients’ anxiety.

Even though our study is based on a large sample, some limitations should be acknowledged: no control group was involved, and our results could hardly be generalized to different geographic populations due to inclusion of patients enrolled in a single institution. Additionally, our multidisciplinary management, with a focus on patient engagement and shared decision-making (Eysenck and Eysenck, 1991), could further influence the generalizability of our results. Of note, compared to a mono-disciplinary approach, our multidisciplinary management provides a dedicated psychologist who offers clinical support on patients’ potential anxiety.

Based on the present findings, and our 15-year experience within a multidisciplinary setting (Lazarus and Folkman, 1984; Eysenck and Eysenck, 1991; Deimling et al., 2017; Marenghi et al., 2017), psychological assessment both at the moment of diagnosis and during AS is needed to detect the risk for increased anxiety. Promoting problem-focused and emotion-focused coping and helping patients in expressing their thoughts and beliefs about cancer and AS are beneficial to the management of anxiety. A multidisciplinary clinical team including the psychologist can promote tailored information, which may help patients in better understanding the choice of AS and, in turn, reduce cancer-related anxiety over time (Eysenck and Eysenck, 1991; Magnani et al., 2012; Deimling et al., 2017). Furthermore, the prognostic nomogram may represent helpful support for the multidisciplinary clinical team because of its specificity and applicability in identifying patients who could suffer from cancer-related anxiety.

In conclusion, QoL of patients in AS is not extensively studied; we know that there are delicate moments and situations (such as PSA measurement, biopsies, and visits) that could influence patients’ well-being. Furthermore, since AS is proposed to avoid the side effects of treatments, it became fundamental to understand how patients live and recognize any critical moments to help them in advance. The present study investigated predictors of anxiety in patients on AS 10 months after diagnosis. Focusing on this timing because anxiety can be critical right before the first re-biopsy (12 months after diagnosis) as it may disconfirm the observational option. An important role of personality traits (i.e., extraversion) and coping strategies (i.e., anxious preoccupation) emerged in our study as predictors of PCa-specific anxiety. Furthermore, men perceptions of physical well-being and the lack of symptoms related to PCa showed the potential to protect against clinical levels of anxiety. Finally, a prognostic nomogram was developed to predict patients’ anxiety at AS entrance, aiming to identify those patients who may need psychological support.

## Data Availability Statement

The datasets presented in this article are not readily available because institutional protocol. Requests to access the datasets should be directed to dataset is not available.

## Ethics Statement

The studies involving human participants were reviewed and approved by Fondazione IRCCS Istituto Nazionale dei Tumori (Milan). The patients/participants provided their written informed consent to participate in this study.

## Author Contributions

MA and PD: project administration, writing original draft, writing review, and investigation. MA, TR, and FB: data curation, analyses, and writing review. BA, NN, LD, TM, CM, JM, VS, ZF, and SR: data collection and writing review. VR and BL: study design, supervision, and writing review. All authors contributed to the article and approved the submitted version.

## Conflict of Interest

The authors declare that the research was conducted in the absence of any commercial or financial relationships that could be construed as a potential conflict of interest.

## Footnotes

1 https://www.R-project.org/

## References

[B1] Alvisi M. F. Repetto C. Rancati T. Badenchini F. Magnani T. Marenghi C. (2018). Italian cultural adaptation of the Memorial Anxiety for Prostate Cancer scale for the population of men on active surveillance. *Tumori* 104 172–178. 10.5301/tj.5000646 28623635

[B2] Anderson J. Burney S. Brooker J. E. Ricciardelli L. A. Fletcher J. M. Satasivam P. (2014). Anxiety in the management of localised prostate cancer by active surveillance. *BJU Int.* 114 (Suppl. 1), 55–61. 10.1111/bju.12765 25070423

[B3] Bangma C. H. Bul M. van der Kwast T. H. Pickles T. Korfage I. J. Hoeks C. M. (2013). Active surveillance for low-risk prostate cancer. *Crit. Rev. Oncol. Hematol.* 85 295–302. 22878262 10.1016/j.critrevonc.2012.07.005

[B4] Bellardita L. Dordoni P. De Luca L. Delor J. P. M. Valdagni R. (2018). “Better-Informed Decision-Making to Optimize Patient Selection,” in *Active Surveillance for Localized Prostate Cancer*, ed. Klotz L. (Cham: Humana Press), 149–167. 10.1007/978-3-319-62710-6_14

[B5] Bellardita L. Rancati T. Alvisi M. F. Villani D. Magnani T. Marenghi C. (2013). Predictors of health-related quality of life and adjustment to prostate cancer during active surveillance. *Eur. Urol.* 64 30–36. 10.1016/j.eururo.2013.01.009 23357351

[B6] Bellardita L. Valdagni R. Van Den Bergh R. Randsdorp H. Repetto C. Venderbos L. D. (2015). How does active surveillance for prostate cancer affect quality of life? A systematic review. *Eur. Urol.* 67 637–645. 10.1016/j.eururo.2014.10.028 25454617

[B7] Bokhorst L. P. Valdagni R. Rannikko A. Kakehi Y. Pickles T. Bangma C. H. (2016). PRIAS study group. a decade of active surveillance in the PRIAS study: an update and evaluation of the criteria used to recommend a switch to active treatment. *Eur. Urol.* 70 954–960. 10.1016/j.eururo.2016.06.007 27329565

[B8] Deimling G. T. Albitz C. Monnin K. Renzhofer Pappada H. T. Nalepa E. (2017). Personality and psychological distress among older adult, long-term cancer survivors. *J. Psychosoc. Oncol.* 35 17–31. 10.1080/07347332.2016.1225145 27541961

[B9] Dordoni P. Badenchini F. Alvisi M. F. Menichetti J. De Luca L. Di Florio T. (2020). How do prostate cancer patients navigate the active surveillance journey? A 3-year longitudinal study. *Support. Care Cancer* [Epub ahead of print]. 10.1007/s00520-020-05524-8 32424643

[B10] Esper P. Mo F. Chodak G. Sinner M. Cella D. Pienta K. J. (1997). Measuring quality of life in men with prostate cancer using the functional assessment of cancer therapy-prostate instrument. *Urology* 50 920–928. 10.1016/S0090-4295(97)00459-7 9426724

[B11] Eysenck H. J. Eysenck S. B. G. (1991). *Manual of the Eysenck Personality Scales (EPS Adult).* London: Hodder & Stoughton.

[B12] Folkman S. Lazarus R. S. (1984). *Stress, Appraisal, and Coping.* New York, NY: Springer Publishing Company 150–153.

[B13] Francis L. J. Brown L. B. Philipchalk R. (1992). The development of an abbreviated form of the Revised Eysenck Personality Questionnaire (EPQR-A): its use among students in England, Canada, the USA and Australia. *Pers. Individ. Differ.* 13 443–449. 10.1016/0191-8869(92)90073-x

[B14] Jylhä P. Isometsä E. (2006). The relationship of neuroticism and extraversion to symptoms of anxiety and depression in the general population. *Depress Anxiety* 23 281–289. 10.1002/da.20167 16688731

[B15] Klotz L. Vesprini D. Sethukavalan P. Jethava V. Zhang L. Jain S. (2015). Long-term follow-up of a large active surveillance cohort of patients with prostate cancer. *J. Clin. Oncol.* 33 272–277. 10.1200/jco.2014.55.1192 25512465

[B16] Klotz L. H. (1997). PSAdynia and other PSA-related syndromes: a new epidemic-a case history and taxonomy. *Urology* 50 831–832. 10.1016/s0090-4295(97)00490-1 9426708

[B17] Lazarus R. S. Folkman S. (1984). “Coping and adaptation,” in *The Handbook of Behavioral Medicine*, ed. Gentry W. D. (New York, NY: Guilford), 282–325.

[B18] Magnani T. Valdagni R. Salvioni R. Villa S. Bellardita L. Donegani S. (2012). The 6-year attendance of a multidisciplinary prostate cancer clinic in Italy: incidence of management changes. *BJU Int.* 110 998–1003. 10.1111/j.1464-410X.2012.10970.x 22404874

[B19] Marenghi C. Alvisi M. F. Palorini F. Avuzzi B. Badenchini F. Bedini N. (2017). Eleven-year management of prostate cancer patients on active surveillance: what have we learned? *Tumori* 103 464–474. 10.5301/tj.5000649 28623636 PMC6379800

[B20] Marzouk K. Assel M. Ehdaie B. Vickers A. (2018). Long-term cancer specific anxiety in men undergoing active surveillance of prostate cancer: findings frokm a large prospective cohort. *J. Urol.* 200 1250–1255. 10.1016/j.juro.2018.06.013 29886089 PMC6705118

[B21] O’Connor A. M. (1995). Validation of a decisional conflict scale. *Med. Decis. Mak.* 15 25–30. 10.1177/0272989x9501500105 7898294

[B22] Palorini F. Rancati T. Cozzarini C. Improta I. Carillo V. Avuzzi B. (2016). Multi-variable models of large International Prostate Symptom Score worsening at the end of therapy in prostate cancer radiotherapy. *Radiother. Oncol.* 118 92–98. 10.1016/j.radonc.2015.11.036 26777123

[B23] Parker P. A. Davis J. W. Latini D. M. Baum G. Wang X. Ward J. F. (2016). Relationship between illness uncertainty, anxiety, fear of progression and quality of life in men with favourable-risk prostate cancer undergoing active surveillance. *BJU int.* 117 469–477. 10.1111/bju.13099 25714186 PMC4547910

[B24] Riggio H. R. Riggio R. E. (2002). Emotional expressiveness, extraversion, and neuroticism: a meta-analysis. *J. Nonverb. Behav.* 26 195–218.

[B25] Roth A. Nelson C. J. Rosenfeld B. Warshowski A. O’shea N. Scher H. (2006). Assessing anxiety in men with prostate cancer: further data on the reliability and validity of the Memorial Anxiety Scale for Prostate Cancer (MAX–PC). *Psychosomatics* 47 340–347. 10.1176/appi.psy.47.4.340 16844894

[B26] Roth A. J. Rosenfeld B. Kornblith A. B. Gibson C. Scher H. I. Curley-Smart T. (2003). The memorial anxiety scale for prostate cancer: validation of a new scale to measure anxiety in men with with prostate cancer. *Cancer* 97 2910–2918. 10.1002/cncr.11386 12767107

[B27] Steginga S. K. Occhipinti S. Gardiner R. A. Yaxley J. Heathcote P. (2004). Prospective study of men’s psychological and decision-related adjustment after treatment for localized prostate cancer. *Urology* 63 751–756. 10.1016/j.urology.2003.11.017 15072894

[B28] Tan H. J. Marks L. S. Hoyt M. A. Kwan L. Filson C. P. Macairan M. (2016). The relationship between intolerance of uncertainty and anxiety in men on active surveillance for prostate cancer. *J. Urol.* 195 1724–1730. 10.1016/j.juro.2016.01.108 26872841 PMC4871722

[B29] Tibshirani R. (1996). Regression shrinkage and selection via the lasso. *J. R. Stat. Soc. Ser. B* 58 267–288. 10.1177/0272989X9501500105 7898294

[B30] Van Buuren S. (2012). *Flexible Imputation of Missing Data.* Boca Raton, FL: Chapman & Hall.

[B31] Van Den Bergh R. C. Essink-Bot M. L. Roobol M. J. Wolters T. Schröder F. H. Bangma C. H. (2009). Anxiety and distress during active surveillance for early prostate cancer. *Cancer* 115 3868–3878. 10.1002/cncr.24446 19637245

[B32] Venderbos L. D. F. van den Bergh R. C. N. Roobol M. J. Schröder F. H. Essink-Bot M. Bangma C. H. (2015). A longitudinal study on the impact of active surveillance for prostate cancer on anxiety and distress levels. *Psychooncology* 24 348–354. 10.1002/pon.3657 25138075

[B33] Villa S. Kendel F. Venderbos L. Rancati T. Bangma C. Carroll P. (2017). Setting an agenda for assessment of health-related quality of life among men with prostate cancer on active surveillance: a consensus paper from a european school of oncology task force. *Eur. Urol.* 71 274–280. 10.1016/j.eururo.2016.09.041 27720532

[B34] Ware J. E. Sherbourne C. D. (1992). The MOS 36-item short-form health survey (SF-36). I. Conceptual framework and item selection. *Med. Care* 30 473–483. 10.1097/00005650-199206000-00002 1593914

[B35] Watson M. Law M. G. Dos Santos M. Greer S. Baruch J. Bliss J. (1994). The Mini-MAC: further development of the mental adjustment to cancer scale. *J. Psychosoc. Oncol.* 12 33–46. 10.1300/J077V12N03_03

